# Frontiers Approaches to the Diagnosis of Thrips (Thysanoptera): How Effective Are the Molecular and Electronic Detection Platforms?

**DOI:** 10.3390/insects12100920

**Published:** 2021-10-09

**Authors:** Amalendu Ghosh, Sumit Jangra, Ralf G. Dietzgen, Wen-Bin Yeh

**Affiliations:** 1 Insect Vector Laboratory, Advanced Centre for Plant Virology, ICAR-Indian Agricultural Research Institute, New Delhi 110012, India; amal4ento@gmail.com (A.G.); sumit.jangra712@gmail.com (S.J.); 2 Queensland Alliance for Agriculture and Food Innovation, The University of Queensland, St. Lucia, Brisbane, QLD 4072, Australia; 3 Department of Entomology, National Chung Hsing University, Taichung City 402, Taiwan; wbyeh@nchu.edu.tw

**Keywords:** artificial neural networks, COI, cryptic diversity, differentiation of thrips species, insect taxonomy, molecular markers

## Abstract

**Simple Summary:**

Thrips are important agricultural and forest pests. They cause damage by sucking plant sap and transmitting several plant viruses. Correct identification is the key for epidemiological studies and formulating appropriate management strategies. The application of molecular and electronic detection platforms has improved the morphological character-based diagnosis of thrips species. This article reviews research on molecular and automated identification of thrips species and discusses future research strategies for rapid and high throughput thrips diagnosis.

**Abstract:**

Thrips are insect pests of economically important agricultural, horticultural, and forest crops. They cause damage by sucking plant sap and by transmitting several tospoviruses, ilarviruses, carmoviruses, sobemoviruses, and machlomoviruses. Accurate and timely identification is the key to successful management of thrips species. However, their small size, cryptic nature, presence of color and reproductive morphs, and intraspecies genetic variability make the identification of thrips species challenging. The use of molecular and electronic detection platforms has made thrips identification rapid, precise, sensitive, high throughput, and independent of developmental stages. Multi-locus phylogeny based on mitochondrial, nuclear, and other markers has resolved ambiguities in morphologically indistinguishable thrips species. Microsatellite, RFLP, RAPD, AFLP, and CAPS markers have helped to explain population structure, gene flow, and intraspecies heterogeneity. Recent techniques such as LAMP and RPA have been employed for sensitive and on-site identification of thrips. Artificial neural networks and high throughput diagnostics facilitate automated identification. This review also discusses the potential of pyrosequencing, microarrays, high throughput sequencing, and electronic sensors in delimiting thrips species.

## 1. Introduction

Thrips are soft-bodied slender insects with fringed wings in the order Thysanoptera with nearly 7700 species and over 1200 genera [1]. The family Phlaeothripidae is the most speciose among the thrips with about 3500 described species followed by Thripidae with 2400 described species [2,3]. The phytophagous thrips cause direct damage by piercing plant tissue and imbibing sap. Thrips feeding makes the plant parts appear silvery as the empty cells are filled with air. As the damaged leaves, flowers, and fruits grow in size, they become scarred, malformed, and distorted [4]. Economic losses due to thrips account for GBP 7–11 million annually in the United Kingdom [5]. In addition, thrips cause indirect damage by transmitting viruses, including tospoviruses, ilarviruses, carmoviruses, sobemoviruses, and machlomoviruses. Tospoviruses are economically damaging to a wide range of food crops and ornamental species [6,7,8], causing stunted growth, formation of chlorotic and necrotic rings, death of apical shoots, dropping of leaves, and may be lethal [7,9,10]. Tomato spotted wilt virus (TSWV, order *Bunyavirales,* and family *Tospoviridae*) alone is reported to cause global economic losses of around USD 1 billion [11]. Groundnut bud necrosis virus (GBNV) reportedly causes economic losses of about USD 89 million in Asia [12]. 

Accurate identification is crucial for discriminating thrips species of quarantine concern from endemic species and in formulating effective pest management strategies. Their small size, cryptic habit, color morphs, secondary sexual characters, and genetic variants render the identification of thrips species challenging [13,14,15,16,17]. Conventional insect taxonomy mostly relies on external morphology-based dichotomous keys for species delimitation. Several such resources are available for the identification of thrips specimens [18,19,20,21,22,23,24,25,26,27,28]. However, species identification based on morphological characters is time-consuming as it involves processing of specimens, preparation of microscope slides, and magnification using a microscope, as well as expert morphological knowledge of the genera. Furthermore, available keys are generally limited to the adult stage, and economically important or prevalent thrips species have been illustrated. Moreover, morphological characters do not take into account the presence of cryptic species or genetic variants.

Advancements in molecular biology over the last decade offer a variety of tools for specific and accurate identification of thrips, alleviating the limitations of morphological key-based identification. Nucleic acid and protein-based techniques such as polymerase chain reaction (PCR), random amplified polymorphic DNA (RAPD), restriction fragment length polymorphism (RFLP), sequence-characterized amplified regions (SCAR) markers, quantitative PCR (qPCR), loop-mediated isothermal amplification (LAMP), and monoclonal antibodies (MAb) [14,29,30,31,32,33,34,35,36,37] have been shown to successfully discriminate between several thrips species. Mehle and Trdan [38] previously reviewed the limitations and advantages of traditional and early modern methods of thrips identification. Since then, many advanced molecular and automated electronic techniques have been introduced to improve efficiency or to add functionality in thrips diagnosis. Despite the limitations of traditional thrips identification and recent molecular advances, routine identification of pest thrips species continues to be based on microscopy by quarantine and agriculture departments in many countries due to its dependability. This review provides an update and comparative assessment of novel molecular, high throughput, and automated approaches for fast and precise thrips diagnosis to study thrips polymorphism and to understand thrips population structure. 

## 2. Landmarks in Thrips Diagnostics

**Figure 1:**
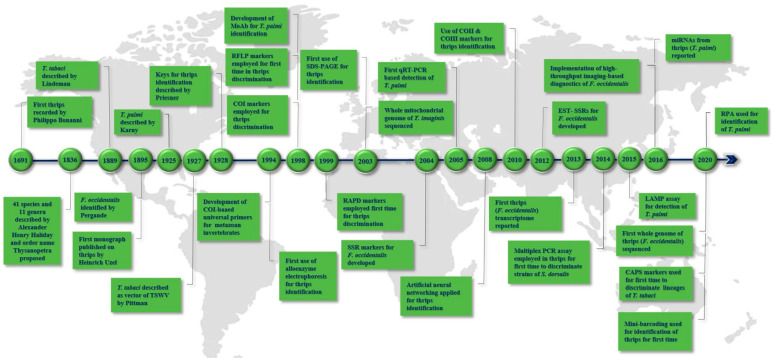
Timeline of milestones in the diagnostics of thrips species.

The first recorded mention of thrips was a sketch by the Italian Jesuit scholar Filippo Bonanni in 1691. Two species in the genus *Physapus* were described by Baron Charles De Geer in 1744, and Linnaeus mentioned a third species in 1746 and named this group as *Thrips*. The insect order Thysanoptera was proposed in 1836, and 41 thrips species and 11 genera were described by Alexander Henry Haliday. The first monograph on thrips was published by Heinrich Uzel in 1895 which was comprised of all the previously published data on thrips and also described 11 new genera and 65 species with their identification keys [39]. Thrips were described as vectors of tomato spotted wilt disease by Pittman in Australia in 1927 [40]. Since then, morphology-based classification of thrips has been advanced globally by many researchers. Thrips became a major concern in agriculture post-1980s due to epidemics of TSWV in North America, Europe, Africa, and Australia following the introduction of the highly efficient vector *Frankliniella occidentalis*. The identification of thrips gained momentum post-1990s following the introduction of mitochondrial cytochrome C oxidase subunit I (COI)-based DNA barcoding. The first protein-based diagnostics were introduced when alloenzyme electrophoresis was used for the identification of thrips species [41]. The first molecular characterization of thrips was reported in the late 1990s [42], and monoclonal antibodies were developed for thrips identification [29]. Molecular identification of thrips was subsequently diversified with the introduction of DNA marker assays. LAMP, multiplex PCR, quantitative PCR, recombinase polymerase amplification (RPA), and high-throughput sequencing were harnessed for thrips diagnostics post 2010s. The use of an artificial neural network system for automated thrips identification was initiated in 2008 [43]. A timeline of landmarks in thrips diagnosis is illustrated in Figure 1. The many advancements in molecular thrips diagnostics, their applications, strengths, and weaknesses are summarized in the subsequent sections. 

## 3. PCR-Based Identification of Thrips Using Molecular Markers

The utility of PCR to analyze small sample sizes insufficient for morphological thrips identification is advantageous for the diagnosis of immature insect stages. Molecular markers are generally highly reliable for resolving species ambiguities that are often not possible with morphology-based taxonomy. In thrips, DNA markers such as COI, COII, COIII, ribosomal RNA (rRNA), internal transcribed spacers (ITS), random amplified polymorphic DNA (RAPD), restriction fragment length polymorphism (RFLP), amplified fragment length polymorphism (AFLP), and simple sequence repeats (SSR) have been utilized successfully for species discrimination and phylogenetic analyses [13,14,44,45,46]. 

### 3.1. COI Markers

**Figure 2:**
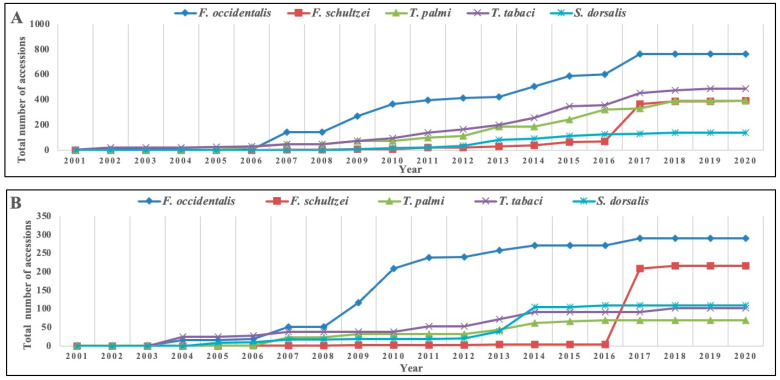
Trend of sequence availability for important thrips species in the NCBI database. (**A**) COI and (**B**) rRNA-ITS sequence accessions. Sequence accessions for five important thrips species, viz., *Frankliniella occidentalis*, *F. schultzei*, *Scirtothrips dorsalis, Thrips palmi*, and *T. tabaci* deposited in the NCBI database were considered. Unverified accessions were removed, and data were processed according to the year of submission. Vertical axis shows the total number of accessions, and horizontal axis represents the year of submission. COI, mitochondrial cytochrome oxidase subunit I; rRNA, ribosomal RNA; ITS, internal transcribed spacer.

The utilization of COI for large-scale DNA barcoding was first proposed in 2003 [47,48]. The first molecular study of thrips based on the partial sequence of COI was reported in 1998 [42]. Since then, more than 14,700 sequences of thrips COI have been deposited in the National Center for Biotechnology Information (NCBI) database to date. The availability of such a large number of reference sequences and robust universal primers [49] has made COI a natural choice for thrips identification. The largest number of COI sequences is available for *Taeniothrips inconsequens* (Uzel) (2171) followed by *Frankliniella occidentalis* (Pergande) (826), *Thrips tabaci* Lindeman (562), *Aptinothrips rufus* (Haliday) (512), *T. palmi* Karny (481), and *F. schultzei* (Trybom) (391). There has been a continuing upwards trend of new thrips COI accessions available in the NCBI database over the years (Figure 2A). A surge of COI data occurred post 2007, but numbers have plateaued in recent years. 

Analysis of COI sequences proved useful in identifying several thrips species infesting economically important crops in southern Africa [50], India [51], and Mexico [52,53]. COI markers are often utilized to substantiate morphological character-based identification [44]. *F*. *occidentalis*, *Haplothrips* spp., *T. palmi*, *T. vulgatissimus* Haliday, and *T. tabaci* could be discriminated based on a 413 bp fragment [54]. A 433 bp fragment of COI was used as a genetic marker for the identification of the ten thrips species including *F. occidentalis*, *Parthenothrips dracaenae* (Heeger), *Anaphothrips obscurus* (Müller), *T. palmi, T. tabaci*, *T. angusticeps* Uzel, *Echinothrips americanus* Morgan, *Hercinothrips femoralis* (Reuter), *H. haemorrhoidalis* (Bouché), and *T. picipes* (Zetterstedt) [32]. These markers detected the immature stages as well as adults, even up to 1:120 dilutions without any cross-reactivity to other thrips species [55,56]. In addition to morphology, COI sequences were used to substantiate the incursion of new species, such as *H. longisensibilis* Xie, Mound, and Zhang in northern Brazil [57]; and *T. parvispinus* (Karny) [58], *Podothrips erami* Minaei, and *F. occidentalis* in India [59,60]. Furthermore, strain-specific COI PCR primers were shown to efficiently discriminate strains and reproductive stages of *T. tabaci* [61]. COI-based identification of thrips species is simple, quick, and reliable. The thrips specimens studied by scanning electron microscopy for morphological keys can also be used as samples for sequencing COI [62]. However, COI sequences of several thrips species demonstrated high intraspecific diversity resulting in a low barcode gap among the species. This can give rise to variation in reference sequence data, reduced efficiency of species-specific primers, and often results in inaccurate identification of species. Nuclear integration of COI fragments is also common in arthropods [63,64,65,66] that may co-amplify or even be amplified instead of mitochondrial COI and negatively affect the molecular identification of thrips. A multi-locus phylogeny is, therefore, recommended for identifying/excluding species ambiguities in thrips.

### 3.2. Thrips Genetic Diversity Studies Using COI Markers

The high sequence variability of 5′-COI has been successfully utilized for identification of genetic variants, reproductive morphs, haplotypes, biotypes, ecotypes, allopatric speciation, subspecies, or cryptic species of several thrips [67]. 

#### 3.2.1. *T. tabaci*

The host preference and tospovirus transmission ability of *T. tabaci* vary considerably between populations. Moreover, *T. tabaci* follows a haplodiploid reproduction with two different reproductive forms, i.e., arrhenotoky and thelytoky [68]. Arrhenotoky produces unfertilized eggs that develop into haploid males, while thelytoky produces diploid females. These two reproductive forms are indistinguishable by morphological keys [69]. Zawirska (1976) [70] proposed the presence of two biotypes of *T. tabaci*. The ‘tabaci type’ prefers tobacco plants and efficiently transmits TSWV. In contrast, populations of the ‘communis type’ neither infest tobacco nor transmit TSWV. However, this hypothesis received little attention until Chatzivassiliou [71] reinforced the concept and demonstrated the heterogeneity of *T. tabaci* populations. The hypothesis that *T. tabaci* is a heterogeneous taxon is supported by abundant variations in its COI region between different populations [32,66,72,73,74]. Based on variation in the COI sequences, three haplotypes were identified at elevated frequencies, of which one represents a high-copy nuclear pseudogene and two are heteroplasmic variants of mitochondrial DNA [66]. Clustering analyses and haplotype networking based on partial COI sequences strongly suggest three major lineages in *T. tabaci* [13,75,76]. Clade ‘T’ or group C exclusively consists of haplotypes collected on tobacco plants, whereas haplotypes collected on leek form a separate clade. The leek clade can be subdivided into ‘L1′ or group A that contains all arrhenotokous strains and male specimens while all thelytokous strains are in group B or ‘L2′ [13,75]. T type arrhenotokous population has a high ability to transmit TSWV, while arrhenotokous L1 are poor transmitters and thelytokous L2 are non-transmitters [75]. An ancient arrhenotokous strain probably differentiated into tobacco (T) and leek (L) types and then a thelytokous type (L2) that originated from the arrhenotokous leek type (L1). Brunner and colleagues described these linages as subspecies in view of genetic distinctiveness in sympatry, as T, L1, and L2 remain distinct both genetically and ecologically [13]. Although Groups A (L1) and B (L2) are monophyletic [75], reproductive isolation will require future in-detail comparisons. Lineages of *T. tabaci* proposed by Brunner and colleagues [13] are widely adopted to describe the divergence in global population of *T. tabaci*. 

All studied Australian *T. tabaci* populations are in L2 lineages [77]. Within this clade the seven populations from potato, three from onion, and four from chrysanthemum, impatiens, lucerne, and blackberry nightshade clustered as three distinct sub-groupings characterized by the source host. The *T. tabaci* population from potatoes is capable of transmitting TSWV [77]. However, New Zealand and Australian populations are different from each other (personal communication, LA Mound). *T. tabaci* males are known to occur in New Zealand, but no male of *T. tabaci* has ever been diagnosed from Australia. All the haplotypes infesting onion and cabbage in the state of New York are L2 [78]. The L1 lineage is also present in New York [79]. L1 and L2 lineages are also recorded in Italy [80]. The occasional presence of arrhenotokous populations in L2 clade may be remnants of the ancestral thrips. The presences of tetraploid females and deuterotoky are evident in *T. tabaci* that may also produce haploid males [17,81].

#### 3.2.2. *T. palmi*

*T. palmi* populations can be divided into two distinct clades based on a high intraspecific variation of COI region [82,83,84,85]. However, in our recent study [86], haplotype data based on COI sequences identified 29 haplotypes of *T. palmi* globally that can be divided into three major clades. The most common haplotype (*n* = 121) is shared among populations from India, Pakistan, Japan, Thailand, Dominican Republic, China, United States, and Taiwan. All Indonesian specimens form a separate haplotype [86]. These haplotypes represent three molecular operational taxonomic units (MOTUs). MOTU refers to clusters of haplotypes that are grouped by DNA sequence similarity of a specific marker gene. Sequences are clustered according to their similarity, and MOTUs are defined based on the similarity threshold. High genetic distances between these MOTUs in *T. palmi* may indicate the presence of cryptic species that are morphologically indistinguishable [87]. 

#### 3.2.3. *Scirtothrips dorsalis* Hood

The heterogeneity of *S. dorsalis* populations has been revealed using COI sequences [14,83,88]. Dickey and colleagues described *S. dorsalis* as a species complex comprised of at least nine cryptic and two morphologically distinguishable species, viz., *S. oligochaetus* (Karny), *S. aff. dorsalis* based on COI hypervariability, and multi-locus phylogeny [89]. Two additional cryptic species in *S. dorsalis* reported by Iftikhar and colleagues considering barcode index numbers (BINs) as species proxies bring the number to 11 [85]. The ‘South Asia 1′ cryptic species is highly invasive, polyphagous, and capable of tospovirus transmission [89]. 

COI-based phylogenetic analysis indicates that the ‘South Asia 1′ cryptic species or C strain [90] was responsible for the first European outbreak of *S. dorsalis* in the Palm House collections at Kew Gardens in 2007 [91]. It has been speculated that strain C arrived in Japan with imports of agricultural commodities from Southeast Asia. The Indian population of ‘South Asia 1′ has the highest genetic diversity with 52 haplotypes and is, thus, considered as the native range of *S. dorsalis* [89]. The narrow host ranges and distributions of ‘South Asia 2′ and ‘East Asia 1′ or YT strain [90] suggest that they are at an earlier stage of global invasion. The other members of the species complex are regionally endemic, varying in their pest status and degree of polyphagy [89].

#### 3.2.4. *F. schultzei*

Two color morphs are known in *F. schultzei*, viz., black/dark and yellow/pale [92]. The black morph is thelytokous, predominant south of the Equator, and the pale morph is arrhenotokous, mainly found in the northern hemisphere [92,93,94]. In India, the Philippines, New Guinea, northern Australia, and East Africa, both forms are abundant in the same ecological niche with overlapping host ranges [87,92,95,96]. The black morph appears to be a better vector of tospoviruses than the pale morph [92,94]. However, pale *F. schultzei* is the major tospovirus vector in field crops of North Queensland, Australia, where both sexes occur (personal communication, LA Mound). Barriers to interbreeding between the color morphs appear to exist [97]. In addition to these two color morphs, a brown color morph of *F. schultzei* has been reported from Australia [98]. Despite their differences in color, these thrips morphs are considered as one species due to similar morphological and ecological features. However, based on COI phylogeny, *F. schultzei* appears to be a species complex [50,99] with three deeply divergent clades corresponding to the three distinct color morphs [87]. The yellow, black, and brown morphs are considered as three different species [98]. Cryptic species IIIa1 corresponds to the dark form, and IIa1 is similar to the pale/yellow form. The yellow form of *F. schultzei* has been identified as a separate species, *F. sulphurea* Schmutz [98]. The description of *F. sulphurea* within *F. schultzei* by the systematists may be due to the co-occurrence with the dark form of *F. schultzei* on the same hosts [96]. The different color morphs of *F. schultzei* are allopatric populations showing genetic divergence that results in reproductive isolation, thereby contributing to speciation.

#### 3.2.5. *F. occidentalis*

Three color morphs are known for *F. occidentalis* [100]. The ‘lupin strain’ causes no apparent damage to crops [101], and a ‘glasshouse’ strain exhibits more resistance to insecticides [101,102]. COI sequence appears useful in confirming the cryptic species status of *F. occidentalis* [84,103]. The haplotypes of *F. occidentalis* clustered in two major lineages. One lineage (HD) that is associated with hot/dry climates corresponds to ‘G’ cryptic species or glasshouse strain; the second lineage (CM) is restricted to cool/moist climates and corresponds to ‘L’ cryptic species or lupin strain [15,16,101,102,104]. These cryptic species may be due to allopatric distribution or ecotypes with partial reproductive isolation. Cryptic species G is predominant in Europe, Africa, Asia, and Australia. Both L and G strains are present in China [104,105,106,107,108]. COI sequences of *F. occidentalis* population in the Netherlands indicate that G is the only strain there [109]. 

COI sequences have also been used to unravel genetic diversity and cryptic species in other important thrips species including *Aeolothrips distinctus* Bhatti, *Franklinothrips megalops* (Trybom), *Mycterothrips nilgiriensis* (Ananthakrishnan), *T. alatus* Bhatti, *T. hawaiiensis* (Morgan), *S. perseae* Nakahara, and *S. oligochaetus* [51,84,87]. Haplotypes of *H. nigricornis* (Bagnall), *H. sylvanus* Faure, and *S. aurantii* Faure have also been identified based on variation in COI sequences [50,110]. *Leucothrips furcatus* Hood has been separated into two clades that show differences in reproductive and feeding behavior [111]. The existence of such a large number of morphologically indistinguishable invasive species raises practical concerns in thrips diagnosis, monitoring, and management. 

### 3.3. COII Markers

Apart from COI, COII is often used for the phylogenetic analysis of insects [112]. A considerable amount of sequence information is available for COII gene of thrips. A total of 305 accessions of COII from different thrips species are available in NCBI, of which 180 sequences account for *A. rufus* followed by *T. palmi* (36) and *F. occidentalis* (30). COII sequences can be used for substantiating COI-based phylogeny of thrips [82]. 

### 3.4. COIII Markers

A total of 82 COIII sequences are available in NCBI for the order Thysanoptera of which the highest number is for *T. palmi* (29). COIII-based phylogeny of *T. palmi* shows a high interspecific distance with no within-species divergence [82]. COIII-based markers appear suitable for the identification of thrips at the genus level but not at the species level. 

### 3.5. rRNA-ITS

In insects, the spacer DNA between 18S and 5.8S RNA genes is known as ITS1, while ITS2 separates genes encoding 5.8S and 28S [113]. Due to unequal crossing-over and gene conversion, nuclear rRNA genes undergo rapid concerted evolution by repairing mismatches among recombining chromosomes. This promotes intragenomic homogeneity of the repeat units and maintains intragenomic uniformity. Moreover, ITS is easy to detect from small quantities of DNA as it is present in high copy numbers. ITS offers additional advantages for species-level identification in thrips due to larger interspecific distances than for COI [82,84,85,86,87]. To date, 3885 rRNA-ITS sequences of thrips can be accessed in NCBI. Most such sequences have been obtained for *F. occidentalis* (409) followed by *F. schultzei* (217), *A. rufus* (137), *S. aurantii* (131), and *S. dorsalis* (111). Figure 2B shows the year-by-year submission rate of rRNA-ITS data for economically important thrips species. 

Ribosomal RNA-ITS markers have been used for detection of *S. dorsalis*, *T. palmi*, *F. tritici* (Fitch), *F. intonsa* (Trybom), *F. cephalica* (Crawford), *H. cahirensis* (Trybom), *Dendrothrips eremicola* Priesner, *Kakothrips pisivorus* (Westwood), *Hydatothrips kassimianus* (Priesener), and *Ceratothripoides claratris* (Shumsher) [46,114,115,116,117,118]. ITS has been used as a natural choice of nuclear marker to substantiate mitochondrial marker-based identification and allow multi-locus phylogenetic analyses of *F. occidentalis*, *F. intonsa*, *F. fusca* (Hinds), *T. tabaci*, and *Megalurothrips distalis* (Karny) [72,119,120]. ITS sequences are also useful in distinguishing cryptic species of *F. schultzei* [97], *S. dorsalis* [121,122], *T. tabaci* [123,124], and *S. aurantii* [110]. However, a high variation among taxa indicates that ITS2 may not be appropriate for assessing intraspecific variation of *T. tabaci* populations [72]. The variation of ITS copies within individuals is also known for some insects [125,126,127]. Analysis of ITS2 data of *S. aurantii* indicates the presence of multiple non-identical copies of spacer sequences [110]. Differences in PCR-amplified product size and the inability to generate a reliable alignment of sequences due to the presence of indels may confound the ITS-based identification of some thrips species [128]. 

### 3.6. Other Marker Genes Used for Thrips Identification

In addition to mitochondrial and rRNA-ITS, other genes including histone H3, elongation factor (EF) 1-α, and cytoskeleton maker α -tubulin have also been used in thrips phylogenetic studies. Histone H3 efficiently determined gene flow from an arrhenotokous form of *T. tabaci* to thelytokous form by confirming the passage of the arrhenotokous male-originated histone H3 gene allele to the F_2_ generation [129]. Histone H3 combined with COI and 28s rRNA reveals that asexuality in *A. stylifer* Trybom and *A. karnyi* John has a genetic basis, while it is governed by endosymbionts in *A. rufus* [130]. Histone H3, α-tubulin, and EF1-α support concatenated phylogenetic analysis of thrips species together with commonly used mitochondrial and nuclear markers [131]. A combined rRNA and EF1α tree is well suited for differentiating *Scritothrips* lineage from *Frankliniella* [45]. Histone H3 and EF1α are also useful for substantiating intra-population and inter-population genetic diversity in sexual and asexual populations of *A. rufus* [132]. However, phylogenetic analysis of *T. palmi, T. nigropilosus* Uzel, and *T. flavus* Schrank using histone H3 shows clear overlaps of interspecific and intraspecific distances without a barcode gap [82]. This suggests that histone H3 may be a useful marker for the identification of discrete genera rather than for species-level studies across a genus [86]. 

### 3.7. SSR/Microsatellite Markers

Microsatellites or simple sequence repeats (SSRs) are repetitive DNA motifs composed of 1–6 bp in both coding and non-coding regions of the genome. SSRs are preferred markers for population genetics studies because of their high polymorphism and abundance, co-dominance, high allelic diversity, and ease of detection by PCR [133,134]. SSRs provide demographic information on founder events, invasion history, local adaptation, allelic fixation index (FST), population size, and gene flow of insect pests. 

Microsatellite markers helped to study the migration pattern of *D. minowai* Priesner, *F. occidentalis,* and *T. palmi* [103,135,136]. *D. minowai* probably originated from multiple regions and gradually separated into two groups. High migration rates indicate gene flow from northeast to southwest China [135]. Populations of *T. palmi* that invaded early show relatively high genetic diversity compared to recently emerged populations. The analysis suggests limited ongoing dispersal and geographical isolation of populations by distance. Greenhouses may play a crucial role in the expansion of *T. palmi* distribution to new areas [136]. The genetic diversity of *F. occidentalis* populations in China, USA, and Kenya indicates a relatively low level of gene flow [106,108,137]. However, a considerable genetic divergence exists in *F. occidentalis* populations between host plant species that suggests low gene flow and possible development of biotypes [137]. An expressed sequence tag (EST) database of *F. occidentalis* has also proven helpful in the development EST-SSRs [105,138,139]. Similarly, six and eleven polymorphic SSR loci have been identified from an enriched genomic library in order to gain better insights into the genetic makeup and migration pattern of *S. perseae* and *T. hawaiiensis* [140,141]. More recently, high-throughput sequencing has been successfully utilized to identify SSRs in *F. occidentalis* and *T. palmi* [136,142,143]. Species-specific markers can also be designed for SSR-based identification of thrips species. 

### 3.8. RFLP Markers

RFLP technique distinguishes individuals based on size differences of restriction fragments of an amplified DNA region generated by a specific or multiple sets of restriction endonucleases. RFLP has been successfully employed to diagnose different species and reproductive and color morphs of thrips as detailed below. 

ITS-RFLP technique has been used to identify important thrips species such as *F. bispinosa* (Morgan), *Pezothrips kellyanus* (Bagnall), *S. citri* (Moulton), *S. dorsalis*, *T. tabaci*, *T. nigropilosus*, *F. occidentalis F. intonsa*, *F. pallida* (Uzel), *F. tenuicornis* (Uzel), and *A. obscurus* without any cross-reactivity [144,145,146]. The restriction pattern with *Alu*I and *Sau3*AI allows unambiguous detection of many thrips species including *F. occidentalis*, *T. palmi*, *T. tabaci*, *T. angusticeps*, *Parthenothrips dracaenae* (Heeger), *A. obscurus*, *E. americanus*, *H. femoralis*, *H. haemorrhoidalis*, and *T. picipes* [32]. Thrips species, viz., *E. americanus*, *F. occidentalis*, *F. tenuicornis*, *Helionothrips aino* (Ishida), *H. spinosus* Wilson, *H. haemorrhoidalis*, *H. femoralis*, *Limothrips cerealium* Haliday, *L. denticornis* (Haliday), *Moundothrips apterygus* Wilson, *P. dracaenae*, *Pseudanaphothrips achaetus* (Bagnall), *Rhipiphorothrips cruentatus* Hood, *Selenothrips rubrocinctus* (Giard), *Sigmothrips aotearoana* Ward, *Suocerathrips linguis* Mound and Marullo, *T. nigropilosus*, *T. physapus* Linnaeus, and *T. tabaci* can be discriminated from each other based on characteristic banding patterns of RFLP [147]. Another RFLP protocol developed by Toda and Komazaki [33] has allowed identification of nine species of thrips from Japanese fruit trees. A similar approach has been used by Rugman-Jones and colleagues for identifying seven species of *Scirtothrips* [35]. The color morphs of *F. schultzei* can also be diagnosed based on ITS-RFLP [97]. COI-based RFLP can efficiently discriminate two reproductive morphs, arrhenotokous and thelytokous, of *T. tabaci* upon the digestion of a 490 bp COI amplicon with EcoO109I [148]. 

The major limitation of PCR-RFLP is the requirement for the specific restriction of endonucleases and the difficulty in identifying specific variations when several SNPs are targeted simultaneously. This limitation may be overcome by mixing two endonucleases in a single reaction. However, double and triple digests in RFLP add higher costs in post-PCR analysis [149]. 

### 3.9. RAPD Markers

The RAPD technique uses random primers in PCR for rapid analysis of polymorphisms in genomic DNA [150]. In the case of thrips, RAPD markers were used for the first time by Klein and Gafni [151] to discriminate three morphotypes of *T. tabaci*. Intraspecific genetic variants of *T. tabaci*, *T. palmi,* and *F. intonsa* were also identified using RAPD [152,153,154]. Using RAPD analysis, the Hungarian thrips population has been divided into two groups, Aeolothripidae (*A. intermedius* Bagnall) and Thripidae (*F. intonsa*, *K. robustus* (Uzel), *Odontothrips confusus* Priesner, *T. dilatatus* Uzel, and *T. tabaci*) [31]. RAPD markers were also used to assess the population structure and inter-population and intra-population variability of *Gynaikothrips uzeli* (Zimmermann) [155]. 

RAPD is often used to complement RFLP and ISSR markers. For example, an analysis based on an RFLP marker followed by RAPD has enabled rapid discrimination of early larval stages of *F. occidentalis* and *F. intonsa* [156]. A combination of RAPD and ISSR markers used to characterize thrips populations in India indicates that RAPD markers are more informative than ISSR markers [157]. The main drawback associated with RAPD is its dominant nature that reduces the information provided by each locus. This loss in information can be compensated by using a larger number of markers [158]. RAPD may also generate unstable and variable amplicons due to the low annealing temperature of the short primers used in PCR. 

### 3.10. AFLP Markers

To overcome the limitations of RAPD and RFLP, amplified fragment length polymorphism (AFLP) has been adopted [159]. AFLP yields a large number of marker loci with an average of 50–100 amplicons per primer pair per sample. Moreover, AFLP is highly reproducible and co-dominant; however, dominant AFLPs are also amplified sometimes [160]. AFLP can also be applied to cDNA and used to study differential gene expression in insects [161].

AFLP markers have been found useful for studying genetic polymorphisms and relationships of *T. tabaci* and *F. occidentalis.* A few unique bands specific to each species may also be helpful in developing species-specific diagnostics [162]. A high level of polymorphism among *F. occidentalis* populations has been detected using AFLP markers that suggest that the population from the Netherlands may have migrated to Beijing, China [163]. AFLP also helps to understand the host-related polymorphisms in *F. occidentalis* populations; a thrips laboratory culture was the most distant from other populations in this analysis [109]. 

Despite being more informative than RAPD and RFLP, AFLP requires more time to complete and uses radioactively labeled primers. The amplified products need to be resolved in polyacrylamide sequencing gels or by using automated genotyping equipment for scoring. Sometimes, the presence of microsatellites in the AFLP loci can make the scoring difficult [160].

### 3.11. SCAR Markers

SCAR markers have been developed to overcome the reproducibility issue of RAPDs [164]. In this technique, the termini of RAPD markers are sequenced to develop longer primers (22–24 nt) for specific amplification of a particular locus. SCAR markers are co-dominant, fast, reliable, and less sensitive to reaction conditions. Once developed, they can be employed to screen large numbers of samples accurately, thus saving time [165]. A SCAR marker specific for *F. occidentalis* has been developed using nine familiar thrips species as controls. The specific SCAR primers amplify a product of 320 bp with no cross-reactivity with 41 other thrips species including *F. intonsa*, *F. tenuicornis*, and *T. tabaci* [36]. 

The major drawback associated with SCAR makers is the need for prior knowledge of DNA sequence. Moreover, the identification of thrips species using SCAR makers is time-consuming and relatively expensive [166]. 

### 3.12. Cleaved Amplified Polymorphic Sequence (CAPS) Markers

The CAPS method utilizes restriction digestion of PCR-amplified DNA fragments using 20–25 bp specific primers. The variation in the size of the digested products determines the level of polymorphism. CAPS is an extension of RFLP that saves time and labor. CAPS does not require steps such as Southern blotting and radioactive detection [167,168,169]. CAPS is highly reproducible, co-dominant, and can be performed with a low quantity of DNA (50–100 ng). CAPS markers can discriminate the three cryptic lineages (L1, L2, and T) of *T. tabaci* based on the SNP in the COI fragment [170]. The main limitation of CAPS markers is that they require sequence data for primer design, and sometimes it is difficult to find polymorphisms because of the small size of the amplified fragment. 

### 3.13. Multiplex PCR

Conventional, single-target PCR is limited to the identification of single thrips species. Multiplex PCR overcomes this limitation through the identification of several thrips species concurrently in a single reaction. This is achieved by the generation of a number of PCR products of specified size obtained using multiple specific primer pairs in a multiplexed reaction [171,172]. Multiplex PCR has become popular in the diagnosis of multiple thrips species since it saves time, cost, and effort compared to other methods. Multiplex PCR can differentiate the novel ‘C’ and native ‘YT’ strain of *S. dorsalis* using ITS2 primers [90]. Multiplex PCR using species-specific ITS2 primers concurrently discriminates *F. occidentalis*, *F. intonsa T. tabaci*, *T. hawaiiensis*, and *T. palmi* without cross-reactivity for up to 22 thrips species [173,174]. Furthermore, fifteen agronomically important thrips species can be detected using species-specific forward and universal reverse primers based on the ITS1 sequence in a multiplex PCR. The assay is specific to the target species with no cross-amplification [175]. A common forward and four species-specific reverse primers based on COI sequences discriminate *T. palmi*, *T. tabaci*, *F. occidentalis*, and *F. intonsa* in a single reaction. Testing of thrips samples from different geographical locations shows reproducibility and specificity of the assay [176]. We have developed a multiplex PCR for simultaneous detection of major thrips species that are virus vectors in India [177]. Four pairs of species-specific primers have been designed based on polymorphisms at the 3′-end of ITS2 and COIII regions. The assay discriminates *T. palmi*, *T. tabaci*, *S. dorsalis*, and *F. schultzei* concurrently in a single reaction without cross-reactivity to other predominant thrips species. The specificity of this multiplex PCR has been validated with a large number of known and unknown samples, and it has been used for the identification of thrip vectors in natural vegetation. 

Despite its popularity for concurrent identification of thrips species, the major limitations of multiplex PCR are self-inhibition among different sets of primers, low amplification efficiency, and different efficiencies on different templates [178]. 

### 3.14. Quantitative Real-Time PCR (qPCR)

A major advancement in PCR-based technologies has been the real-time monitoring of specific target amplification through measuring fluorescence signals [179,180]. Among the available quantitative PCR platforms, probe-based qPCR is highly sensitive and specific, and its fluorescently labeled hydrolysis probes can provide multiplexing of reactions [181]. A qPCR-based assay can detect *T. palmi* within 45 min in a mix of 22 thrips species with high specificity [34]. The use of a COI-based Taqman^™^ probe (Applied Biosystems; Thermo Fisher Scientific, Waltham, MA, USA) can efficiently detect *T. palmi* by qPCR. This qPCR assay discriminates *T. palmi* from 11 other thrips species and 12 other members of the family *Thripidae* [182]. Taqman probe-based qPCR has also been used by Huang and colleagues for rapid and sensitive detection of immature stages of *F. occidentalis* [183]. The assay can detect *F. occidentalis* with high specificity and does not cross-react with *F. schultzei*, *F. intonsa*, *S. dorsalis*, *T. tabaci*, and *Caliothrips fasciapennis* (Hinds). The efficiency of this qPCR is 95%, and the sensitivity is 0.1 pg µL^−1^. Przybylska and colleagues [184] have developed a duplex qPCR based on the 5.8S ITS2 ribosomal region to discriminate between *T. palmi* and *F. occidentalis*. This sensitive assay can detect 1 pg of template DNA. A probe-based real-time PCR assay using crude DNA has been shown to detect different thrips species including *F. fusca, F. occidentalis*, *F. tritici*, *T. tabaci*, and *Neohydatothrips variabilis* (Beach) in as little as 1 pg of thrips DNA [185]. However, the operating cost of qPCR is significantly higher than conventional PCR, and it requires prior sequence knowledge to design primers and probes. This technique can only be utilized for diagnosis of thrips species if sufficient genetic information is known [186]. 

### 3.15. Loop-Mediated Isothermal Amplification (LAMP)

LAMP is an isothermal nucleic acid amplification technique that serves as an alternative to PCR [187]. It is performed at a constant temperature of 60–65 °C using either two or three pairs of primers and a DNA polymerase with high strand displacement activity. The sensitivity and specificity of LAMP are better than conventional and nested PCR [188,189]. 

A LAMP assay has been developed to detect *T. palmi* using a real-time platform or a heating block [37]. Real-time LAMP can detect 2 × 10^−4^ adult or 2 × 10^−3^ larval specimens. Using a heating block, products visualized using EvaGreen^®^ dye (Biotium, Fremont, CA, USA) representing 2 × 10^−10^ adult or 2 × 10^−8^ larval specimens could be detected. The assay is faster than other methods of thrips diagnosis as it takes only 30 min to complete [37]. *T. tabaci* can also be detected by LAMP and visualized by appearance of a yellowish-green fluorescence when MnCl_2_ and calcein dye are added. The assay is sensitive and specific to *T. tabaci* without cross-reactivity with other thrips species [190]. 

LAMP has not been utilized to its full potential for diagnosis of thrips. One of the reasons may be the complexity in designing three sets of primers without obtaining cross-reactivity. This also limits the development of multiplexed LAMP assays for identification of more than one thrips species concurrently [191]. 

### 3.16. Recombinase Polymerase Amplification (RPA) 

Most of the reported molecular diagnostics require sophisticated laboratory equipment and cannot be performed on-site in the field. The recently developed RPA is an exception; it is an isothermal assay that can be used for field-based diagnosis [192]. RPA utilizes three core enzymes, recombinase, single-strand binding protein (SSB), and strand-displacement polymerase, for the amplification of target DNA [193]. RPA has been successfully applied for the detection of several animal and plant pathogens [194,195,196,197,198]. We have developed an RPA protocol for field-based identification of *T. palmi* [199]. This RPA is performed with crude extract from a single *T. palmi* in sterile distilled water. The assay can be completed within 20 min at human body temperature by holding the reaction tubes in the hand. The assay is further simplified by adding a fluorescent or colorimetric dye to the reaction mix, thus eliminating the gel electrophoresis step. The presence of *T. palmi* can be visualized by the appearance of fluorescence or by a change in color from dark blue to sky blue. The RPA assay is highly sensitive; it can detect as little as 0.2 ag of target DNA which is 10^9^-fold more sensitive than PCR using the same primers [199]. This on-site, rapid in-field assay for diagnosis of *T. palmi* is easy to use by non-expert personnel in plant biosecurity and pest management. RPA can also be combined with colloidal gold nanoparticles as probes for sensitive detection on disposable screen-printed carbon electrodes [200,201]. 

### 3.17. Protein-Based Diagnostic Assays

Protein-based diagnostics include protein electrophoresis, immune-enzymatic methods, and protein sequencing. Isoenzyme electrophoresis has been extensively used for thrips identification for the past 25 years [202,203]. Alloenzyme electrophoresis has been useful in a population dynamics study of *T. inconsequens* in New England, USA [41]. Eight enzymes have been resolved and stained for three thrips species, viz., *F. occidentalis*, *L. cerealium,* and *T. inconsequens*. Esterase and peptidase are resolved into three distinct bands and provide a reliable basis for species separation. A major limitation associated with allozyme electrophoresis is quick deterioration of enzyme activity if samples are not kept alive or deep frozen immediately after collection [33]. 

A monoclonal antibody (MAb) is available for the serological detection of *T. palmi* [29]. This MAb may be useful for high-throughput screening, but its specificity has been validated only with *T. tabaci* and *F. occidentalis* [29]. Immature stages of *H. haemorrhoidalis* can be detected using sodium dodecyl sulfate-polyacrylamide gel electrophoresis (SDS-PAGE) [204]. A low intraspecific variation and the stability of the protein profile make this technique a valuable tool to diagnose thrips species. Distinct banding patterns in SDS-PAGE have enabled the discrimination of larvae of *T. tabaci* and *F. occidentalis*. SDS-PAGE is also useful for discriminating insecticide-resistant and insecticide-susceptible populations of *F. occidentalis* [205]. The acrinathrin-resistant and methiocarb-resistant populations show higher esterase activity in SDS-PAGE analysis. This method of thrips diagnosis demands careful control of critical aspects of electrophoresis and staining procedure for accurate species identification. 

### 3.18. Mini-Barcode Pyrosequencing

COI at a fragment length of around 650 bp is the most commonly used means to identify insect species by DNA barcoding. Amplification of full-length barcode sequences is time-consuming while dealing with quarantine specimens [206]. Sanger sequencing which is generally used to obtain marker sequences is time-consuming since multiple steps are involved. To overcome this hurdle, mini-barcode pyrosequencing from short COI and 16S rDNA fragments has been used [206]. Pyrosequencing is a single-step ‘sequencing by synthesis’ method that relies on the detection of pyrophosphate release and the generation of light upon nucleotide incorporation [207]. Mini-barcoding by pyrosequencing has been used for species-level identification of *Thrips* spp. [208]. A universal primer pair for the 16S rDNA gene yields an amplicon of 120 bp. The analysis shows interspecies divergence of 4% between *T. tabaci* and *T. palmi*. DNA mini-barcodes are accumulating quickly for more thrips species and may become a popular diagnostic tool in the future. 

### 3.19. Microarray/DNA Chip

Microarrays can detect the expression of hundreds or thousands of genes at the same time. DNA chips are printed with tiny spots in defined positions, and each spot acts as a probe to detect gene expression by hybridization to target sequences. Microarray technology has been extensively used in disease forecasting, diagnostics, screening of drugs, crop improvement, food safety analysis, and environmental monitoring [209,210,211]. DNA chips are preferred in insect diagnosis because of their high throughput, high sensitivity, and many practical applications [212,213]. To date, only two studies report the use of microarray/DNA chip technology in thrips identification. A DNA chip, based on 12 motifs of COI has been used to identify three species of *Frankliniella* [214]. A microarray using ITS1 primer-based probes has been shown to detect 15 thrips species [175]. Based on microarray techniques, a biochip for the simultaneous identification of hundreds of important thrips species could be developed.

### 3.20. Artificial Neural Networks (ANN)

As a component of artificial intelligence (AI), ANN makes the identification of biological objects automated and simple [215]. Combined with statistical tools such as principal component analysis (PCA) or classification trees, these networks meet the standards of science in the digital era. ANN-based web tools or software transform metadata through multilayer system processing that can be utilized in insect species diagnosis. 

ANN has been successfully implemented in the semi-automated identification of 18 European thrips species in four genera, *Aeolothrips*, *Chirothrips*, *Dendrothrips*, and *Limothrips*. Seventeen morphometric and two qualitative two-state characters from head, pronotum, forewing, and ovipositor of thrips and sex are considered as input data. A simple ANN architecture simultaneously detects both males and females of all 18 species in an independent test with 97% accuracy [43]. Similarly, an ANN has been developed that can identify 101 European thrips species with 95% reliability [216]. A three-layer ANN using seventeen morphological and 15 quantitative morphometric variables can discriminate two similar thrips species, *T. sambuci* Heeger and *T. fuscipennis* Haliday, with 100% accuracy [217]. 

An interactive, matrix-based computer diagnostics and information system has been developed by Mound and colleagues [218] in order to facilitate rapid identification of thrips species. Computerized key-based photomicrographs and videos of thrips species allow a user to address any character [23,219]. Such image repositories could be combined with neural networking to standardize a fast and accurate system of thrips identification. Such a combination of image processing and neural networking helps to identify *F. occidentalis* in greenhouses with a high level of precision [220,221]. 

ANN-based identification of thrips is a big step forward in thrips diagnostics, but more refinement is required to make the models more precise and commercially viable. Identifying the immature stages using ANN remains a challenge for its wider application. 

### 3.21. High-Throughput Imaging-Based Diagnostics

International trade and exchange of plant materials have contributed to an increased spread of pests including thrips. Implementation of robust, quick, and accurate detection methods is necessary to combat this situation. High-throughput diagnosis involves analyses of imaging data to detect invasive species. Image processing algorithms based on size, morphological features (shape and boundary), and color have been utilized to identify thrips in glasshouses [222]. Image processing in combination with neural networks can quantify *F. occidentalis* trapped on sticky pads in field and greenhouse conditions with a high precision rate of 92% [220]. Support vector machine (SVM)-based image processing can detect the infestation of thrips in strawberry fields. A mobile robot moves across the field and captures images that are processed online for detection of pests. The system detects the target thrips species with an error rate of less than 2.5% [223]. Recently, high-throughput phenotyping has been used to identify host plant resistance to *F. occidentalis* [224]. High-throughput phenotyping measures the feeding damage caused by thrips using ImageJ and Ilastik software [225]. These automated, high-throughput technologies can also be standardized for species level identification of thrips. The software needs to be coded to process the images into key morphological characters in order to identify the thrips species present in the fields or large shipments.

### 3.22. High-Throughput Sequencing (HTS) 

HTS enables automated de novo sequencing of large amounts of DNA or RNA in a time-effective and cost-effective manner. In thrips, HTS has unveiled the mitochondrial genomes (mitogenomes) of *T. imaginis* Bagnall, *F. occidentalis*, *F. intonsa*, *S. dorsalis*, *A. obscurus*, *T. palmi*, *O. loti* (Haliday), and *N. samayunkur* (Kudo) [226,227,228,229,230,231,232,233]. The transcriptomes of *F. occidentalis* [234,235,236,237], *F. fusca* [238,239], *T. palmi* [240], *T. tabaci* [241], and *F. tritici* [239] have been revealed through HTS. Furthermore, regulatory microRNAs have been identified for *T. palmi* and *T. tabaci* [242,243]. Recently, the complete genomes of *F. occidentalis* and *T. palmi* have been sequenced and annotated [244,245]. The availability of thrips complete mitochondrial and nuclear genome data will support the study of genetics, epigenetics, virus transmission, insecticide resistance, migration pattern, and elucidate many novel facts of thrips biology. In diagnostics, the availability of genome information is useful for identifying novel markers for rapid and precise identification of thrips. Small RNA data will help in the identification of highly specific microRNAs for thysanoptera pest management. The transcriptome data facilitate the identification of key candidate genes involved in virus transmission, insecticide resistance, insect development, and physiological processes. ‘Omics’ of thrips will be helpful in designing genome editing methods to develop thrips-resistant plants. 

## 4. Strengths of the Present-Day Molecular and Electronic Platforms and Future Potential

The limitations of manual morphological identification have been described in this review. Several molecular and electronic detection techniques such as nucleic acid or protein-based assays, enzymatic hydrolysis, ANN, and high throughput imaging-based diagnosis offer valuable alternatives in situations where correct identification is very tedious, time-consuming, or virtually impossible by traditional morphometrics. A list of molecular techniques that have been applied for thrips diagnosis is provided in Table 1.

PCR and direct sequencing have become popular for discriminating thrips species. COI has been preferred over other gene markers due to its wide acceptance as a universal barcode and availability of robust universal primers. However, high intraspecific variability [86] and nuclear introgression of COI fragments of thrips [66] negatively affect molecular identification based on COI. A multi-locus phylogeny should be adopted to increase the precision of molecular marker-based identification of thrips species. 

rRNA-ITS may be a good choice of nuclear marker to generate concatenated phylogeny. Very few other genes have been studied for their potential use as markers in thrips identification. Recently available genome and transcriptome data for several thrips species need to be carefully mined to identify novel marker genes that can be utilized for a more precise diagnosis. 

Polymorphisms are common in thrips species, a fact that is often difficult to describe by classical taxonomy. Climate change may also have significant effects on the diversification of thrips species. Different thrips species may adapt differentially to changing climatic conditions as in the case of *F. occidentalis* [16] or hosts as in the case of *T. tabaci* [13,70]. The existence of such a large number of morphologically indistinguishable entities poses challenges in detection, forecasting of invasion, range expansion, and formulation of effective management strategies. The COI marker shows strong potential in the detection of genetic polymorphisms within species and in resolving species complexes that were thought to be due to intraspecific variation but are actually species-specific attributes. *S. dorsalis* has been found to consist of at least three genetically separable groups that are morphologically indistinguishable [14]. The pale form of *F. schultzei* is actually a separate species, *F. sulphurea* [98]. *F. occidentalis* consists of two sympatric cryptic species ‘G’ and ‘L’ with reproductive isolation [16]. Considerable genetic differences and reproductive isolation among thelytokous and arrhenotokous forms of *T. tabaci* indicate the existence of separate species like *T. nigropilosus* [246]. An in-depth study is needed to explore additional morphological characters specific to different cryptic species. Future studies on species demarcation of thrips should include a larger number of specimens collected across various ecological niches. Information on host preference, temporal occurrence, reproductive isolation, virus transmission, insecticide resistance, microbial endosymbionts, population biology, and other ecological aspects need to be integrated with genetic divergence and gene flow studies to resolve any disputes. The availability of additional genome and transcriptome data of intraspecies biological and ecological variants may provide better clarity on thrips speciation.

The implementation of ANN shows potential to discriminate thrips species with high accuracy [216,217]. SVM-based image processing can be used for high-throughput diagnosis of thrips species [223]. The success of these techniques relies on active collaboration across the field of morpho-taxonomy and robotics. The image repository of thrips needs to be augmented with more species and unique morphological characters to make the automated diagnosis system more precise. However, image-based descriptions of the immature developmental stages are currently limited to only a few thrips species [247,248]. Diagnosis of egg, larval, and pupal stages of thrips species intercepted in imported plant materials is crucial and challenging. Recently developed RPA and LAMP-based thrips diagnostics can help to detect new invasions by the identification of eggs and immature stages. These techniques are highly sensitive and can be completed within 30 min, an ideal choice in plant biosecurity at the port of entry and land border crossings. However, diagnosis becomes complex for a mixed population of thrips species. The implementation of microarrays may be useful in this context. 

Cutting-edge technologies such as electronic sensing can also be applied for automated rapid diagnosis of thrips species. An electronic nose has been successful in monitoring the infestation of spiders and mites in tomatoes [249] and the green vegetable bug *Nezara viridula* (Linnaeus) in cotton [250]. The specific olfactory cues released by different thrips species need to be characterized and exploited in order to develop sensor arrays and pattern recognition systems. The biosensor connects with the associated signal processors and displays the results in a user-friendly manner [251]. Species-specific MAbs targeted against thrips can be used in immune sensors. Nucleic acid-based biosensors may also be developed for thrips species-specific conserved unique sequences. Complementary sequences can be synthesized, labeled, and immobilized on the sensor. The presence of particular thrips species can be ascertained in a user-friendly manner by detecting fluorophore-labeled hybridization. Black phosphorus nanoparticle-based highly sensitive DNA nano-biosensors [252] may also be developed for the detection of thrips. A universal platform based on polypentafluorophenyl acrylate-grafted surfaces is available for the detection of viruses [253]. Such a universal platform with enhanced sensitivity may also be developed for the detection of thrips species. The success of these modern diagnostic techniques will help in the timely identification and management of invasive thrips species in order to protect crops and the environment. 

## Figures and Tables

**Table 1:** List of molecular diagnostics reported for different thrips species.

Serial No.	Molecular Marker/Technique	Thrips Species Detected	References
1.	Mitochondrial cytochrome C oxidase subunit I (COI)	*Aeolothrips distinctus*, *Anaphothrips obscurus*, *Echinothrips americanus*, *Franklinothrips megalops*, *Frankliniella occidentalis*, *F. schultzei*, *F. serrata*, *Haplothrips clarisetis*, *Heliothrips longisensibilis*, *H. haemorrhoidalis*, *H. sylvanus*, *Hercinothrips femoralis*, *Leucothrips furcatus*, *Mycterothrips nilgiriensis*, *Pathenothrips dracaenae*, *Podothrips erami*, *Scirtothrips aurantii*, *S. oligochaetus*, *S. perseae*, *Synaptorthrips psoraleae*, *Thrips alatus*, *T. angusticeps*, *T. hawaiiensis*, *T. palmi*, *T. parvispinus*, *T. tabaci*, *T. vulgatissimus*, *T. picipes*	[13,14,15,16,17,32,44,50,51,52,53,54,55,56,57,58,59,60,61,66,72,73,74,75,76,78,80,81,82,83,84,85,86,87,88,89,90,91,98,99,106,107,108,109,110,111]
2.	COII	*T. palmi*	[82]
3.	COIII	*T. palmi*	[82]
4.	Internal transcribed spacers (ITS)	*Ceratothripoides claratris*, *Dendrothrips eremicola*, *F. cephalica*, *F. fusca*, *F. intonsa*, *F. occidentalis*, *F. schultzei*, *F. tritici*, *H. cahirensis*, *Hydatothrips kassimanus*, *Kakothrips pisivorus*, *Megalurothrips distalis*, *S. aurantii*, *S. dorsalis*, *T. palmi*, *T. tabaci*	[46,72,97,110,114,115,116,117,118,119,120,121,122,123]
5.	Other molecular markers (H3, EF 1-α, and α-tubulin)	*A. karnyi*, *A. stylifer*, *A. rufus*, *Frankliniella* spp., *Scirtothrips* spp., *T. flavus*, *T. nigropilosus*, *T. palmi*, *T. tabaci*, several species in the Melanthripidae, Merothripidae, Phaleothripidae	[45,82,129,130,144]
6.	Single sequence repeat (SSR) or microsatellite markers	*D. minowai*, *F. occidentalis*, *S. perseae*, *T. hawaiiensis*, *T. palmi*	[103,105,106,135,137,138,139,140,141,142,143]
7.	Restriction fragment length polymorphism (RFLP) markers	*A. obscurus*, *E. americanus*, *F. bispinosa, F. intonsa, F. occidentalis*, *F. pallida*, *F. schultzei*, *F. tenuicornis*, *F. tenuicornis, H. spinosus*, *H. aino*, *H. haemorrhoidalis*, *H. femoralis*, *H. femoralis*, *Limothrips denticornis*, *L. cerealium*, *Moundothrips apterygus*, *Pezothrips kellyanus*, *Pathenothrips dracaenae*, *Pseudanaphothrips achaetus*, *Rhipiphorothrips cruentatus, S. aceri, S. astrictus, S. aurantii, S. bounites, S. citri, S. derpanofortis, S. dorsalis, S. frondis, S. inermis, S. kenyensis, S. oligochaetus, S. pan, S. perseae,* spp., *S. rubrocinctus, S. aotearoana, S. linguis,* *T. angusticeps*, *T. coloratus*, *T. flavus*, *T. hawaiiensis*, *T. nigropilosus,* *T. palmi*, *T. physapusm, T. setotus*, *T. tabaci*, *T. picipes*	[32,35,97,144,145,146,147,148]
8.	Random amplified polymorphic DNA (RAPD) markers	*A. intermedius, F. intonsa, F. occidentalis, F. schultzei, Gynaikothrips uzeli, K. robustus, Odontothrips confusus,S. dorsalis*, *T. dilatatus, T. palmi,* and *T. tabaci*	[31,151,152,153,154,155,156,157]
9.	Amplified fragment length polymorphism (AFLP) markers	*F. occidentalis, T. tabaci*	[109,162,163]
10.	Sequence characterized amplified region (SCAR) markers	*F. occidentalis*	[36]
11.	Cleaved amplified polymorphic sequences (CAPS) markers	*T. tabaci*	[170]
12.	Multiplex polymerase chain reaction (PCR)	*F. intonsa, F. occidentalis, F. schultzei, S. dorsalis, T. hawaiiensis, T. palmi, T. tabaci*	[90,173,174,175,176,177]
13.	Quantitative real-time PCR (qPCR)	*F. fusca*, *F. occidentalis, F. schultzei, F. tritici, Neohydatothrips variabilis, T. palmi, T. tabaci*	[34,56,182,183,184,185]
14.	Loop-mediated isothermal amplification (LAMP)	*F. occidentalis, T. palmi*	[37,190]
15.	Recombinase polymerase amplification (RPA)	*T. palmi*	[199]
16.	Protein-based diagnostics	*F. occidentalis*, *H. haemorrhoidalis, L. cerealium, Taeniothrips inconsequens,T. palmi, T. tabaci*	[29,33,41,204,205]
17.	Mini barcoding	*T. palmi, T. tabaci*	[208]
18.	Microarray/ DNA chip	*Dichromothrips smithi, Microcephalothrips abdominalis, F. williamsi, F. cephalica, F. occidentalis, F. intonsa, T. palmi, T. florum, T. hawaiiensis, T. alliorum, T. tabaci, T. fuscipennis, M. usitatus, Stenchaetothrips biformis, S. dorsalis,*	[175,214]
19.	Artificial Neural Networking (ANN)	*A. albicinctus*, *A. astutus, A. ericae, A. fasciatus, A. intermedius, A. versicolor, A. vittatus, Chirothrips aculeatus, C. ambulans, C. hamatus, C. manicatus, C. pallidicornis, D. degeeri, D. ornatus, D. saltatrix, F. occidentalis, L. cerealium, L. consimilis, L. denticornis, T. fuscipennis, T. sambuci*	[43,216,217,218,220,221]
20.	High-throughput imaging-based diagnostics	*F. occidentalis*	[220,221,222,223,224,225]
21.	High throughput sequencing (HTS) (mitogenome andtranscriptomewhole genome)	*A. obscurus*, *F. occidentalis*, *F. fusca, F. intonsa*, *F. tritici, N. samayunkur, O. loti, S. dorsalis*, *T. imaginis*, *T. palmi*, *T. tabaci*	[226,227,228,229,230,231,232,233,234,235,236,237,238,239,240,241,242,243,244,245]

## Data Availability

Not applicable.
